# Spatial Geographic Mosaic in an Aquatic Predator-Prey Network

**DOI:** 10.1371/journal.pone.0022472

**Published:** 2011-07-20

**Authors:** Johel Chaves-Campos, Steven G. Johnson, C. Darrin Hulsey

**Affiliations:** 1 Department of Biological Sciences, University of New Orleans, New Orleans, Louisiana, United States of America; 2 Department of Ecology and Evolutionary Biology, University of Tennessee, Knoxville, Tennessee, United States of America; University of Texas, United States of America

## Abstract

The geographic mosaic theory of coevolution predicts 1) spatial variation in predatory structures as well as prey defensive traits, and 2) trait matching in some areas and trait mismatching in others mediated by gene flow. We examined gene flow and documented spatial variation in crushing resistance in the freshwater snails *Mexipyrgus churinceanus*, *Mexithauma quadripaludium*, *Nymphophilus minckleyi*, and its relationship to the relative frequency of the crushing morphotype in the trophically polymorphic fish *Herichthys minckleyi*. Crushing resistance and the frequency of the crushing morphotype did show spatial variation among 11 naturally replicated communities in the Cuatro Ciénegas valley in Mexico where these species are all endemic. The variation in crushing resistance among populations was not explained by geographic proximity or by genetic similarity in any species. We detected clear phylogeographic patterns and limited gene flow for the snails but not for the fish. Gene flow among snail populations in Cuatro Ciénegas could explain the mosaic of local divergence in shell strength and be preventing the fixation of the crushing morphotype in *Herichthys minckleyi*. Finally, consistent with trait matching across the mosaic, the frequency of the fish morphotype was negatively correlated with shell crushing resistance likely reflecting the relative disadvantage of the crushing morphotype in communities where the snails exhibit relatively high crushing resistance.

## Introduction

Prey often vary in defenses that reduce their susceptibility to predators [1]–[3]. Likewise, predators can show variability in predatory structures. This phenotypic variation provides the substrate for reciprocal evolution between predator and prey at the population level. The amount of reciprocal evolution can also change geographically in its level of intensity especially if geographic subdivision exists and if interactions and their consequences vary from one locality to another [4]–[9]. The “geographic mosaic theory of coevolution” [6] proposes that the dynamics of predator-prey interactions should drive interspecific selection, predator genotype by prey genotype by environment interactions, and trait remixing as a product of microevolutionary forces. The theory predicts several patterns if selection varies geographically, two of which are the focus of this study. First, spatial variation in predatory structures and prey defensive traits should exist. Second, matching of these traits between predators and prey in populations where reciprocal selection is strong, and trait mismatching in populations where selection is weak [6], [10]–[12]. Gene flow, genetic drift, and the dynamics of extinction and recolonization are expected to cause remixing of coevolving traits among populations, making the predictions dependent on the scale of the interaction and the degree of intermixing [6], [9], [10], [13], [14]. Currently, empirical examples of patterns that are consistent with the processes critical to the geographic mosaic theory come from a small number of well-studied and mostly terrestrial systems [11]. Here we document spatial variation in traits that potentially mediate antagonistic interactions in an aquatic predator-prey network and test whether this system exhibits patterns consistent with the geographic mosaic theory of coevolution at a spatial scale that encompasses the entire geographic range of all interacting species.

**Figure 1:**
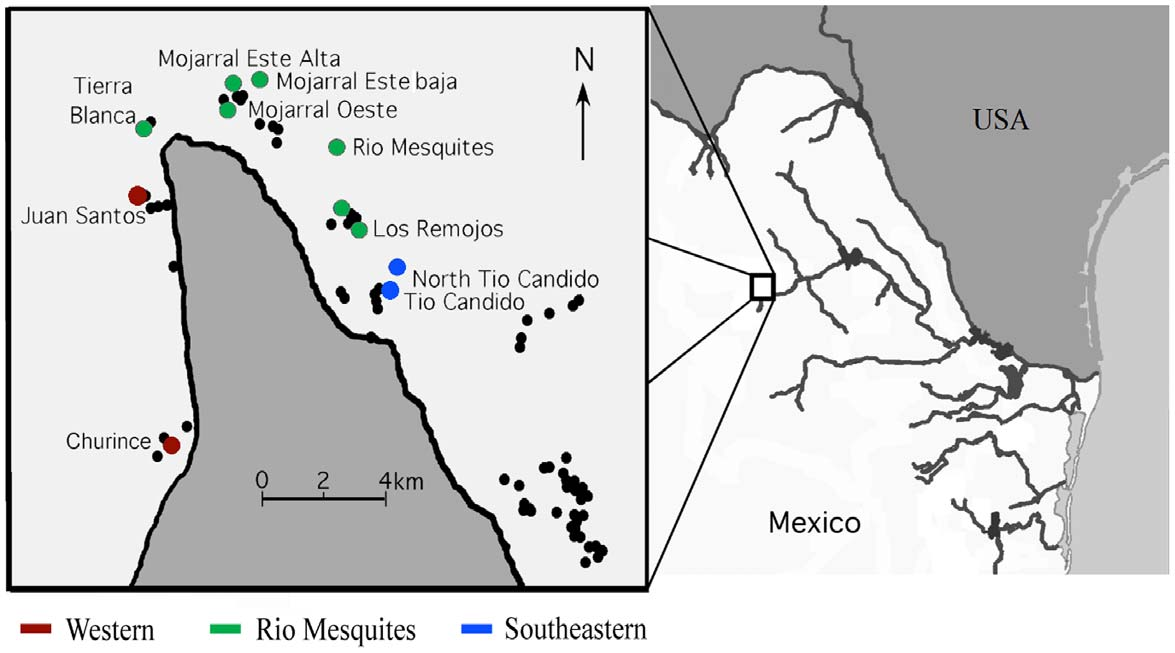
The Cuatro Ciénegas valley in northeastern Mexico and the collecting sites of the three snail species and the cichlid from spring-fed habitats in the valley. These pools and streams are arrayed around a mountain (Sierra de San Marcos) that juts into the center of the valley. Three geographic drainages are presented and color coded: western, Río Mesquites, and southeastern. Small black circles represent unsampled pools in the area. The valley is depicted as inset to the image on the right of the map that shows the boundary between Mexico and the United States.

The geographic mosaic of coevolution theory has largely been studied in pair-wise interactions between a predator and a particular prey across a subset of their geographic range. However, predator-prey interactions normally occur in networks of several interacting species and the number of predator and prey species in these networks can often change substantially from community to community [15]. Herein, we examine a network composed of a fish predator and three snail prey species that are all present in replicated communities across their shared but limited geographic ranges. The freshwater snails *Mexipyrgus churinceanus, Mexithauma quadripaludium*, and *Nymphophilus minckleyi*, as well as their polymorphic fish predator *Herichthys minckleyi,* are all endemic to the small (1500 km^2^) isolated Cuatro Ciénegas valley in the Chihuahuan desert in Mexico [16]–[18]. All four species co-occur in more than 200 springs, spring-fed pools, rivers and playa lakes that exist in the Cuatro Ciénegas basin [19]. These bodies of water are naturally grouped into a few drainages that are not currently connected. Several studies on fully aquatic species have shown that migration is very limited among these drainages but not within drainages, although specific levels of genetic differentiation at the population level have not yet been estimated [20]–[22]. Major isolation in terms of gene flow occurs between drainages located on the western and eastern side of the Sierra de San Marcos (hereafter Sierra) that bisects the Cuatro Ciénegas basin, and between these drainages and the Río Mesquites drainage located close to the tip of the Sierra (Fig. 1) [22]. Hence, these systems could provide evolutionarily independent comparisons across naturally replicated communities with different degrees of genetic isolation.

**Figure 2:**
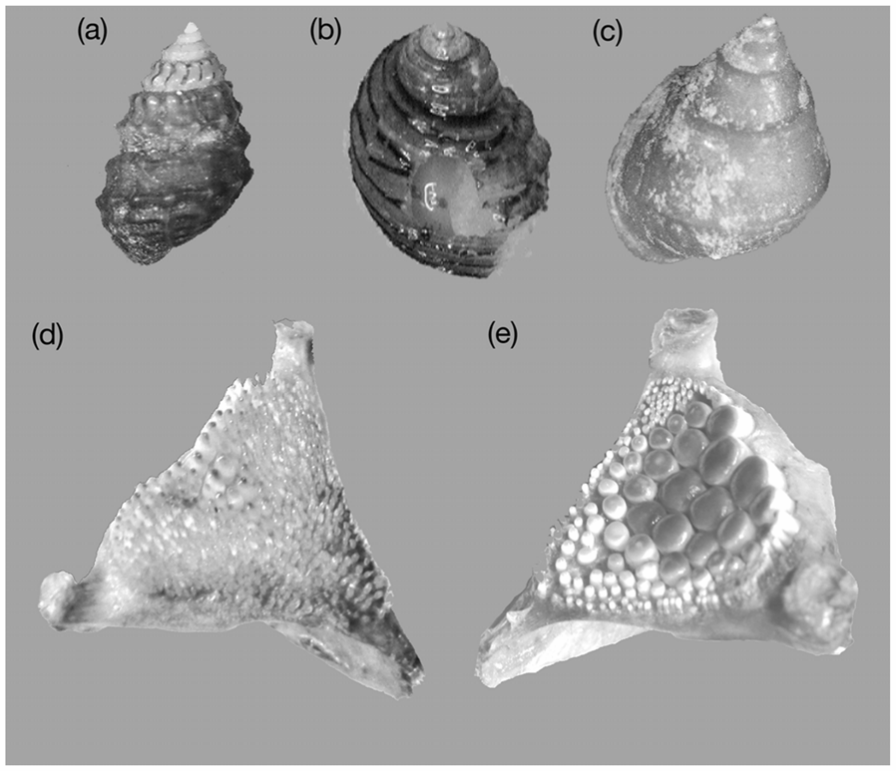
Three hydrobiid snails, *Mexipyrgus churinceanus* (a), *Mexithauma quadripaludium* (b), and *Nymphophilus minckleyi* (c), as well as the papilliform (d) and molariform (e) lower pharyngeal jaw morphotypes of the cichlid fish *Herichthys minckleyi*.

The shells of the snails and jaws of the cichlid appear to be coevolved. The three snails species have sculptured, robust, highly durable shells that are more characteristic of marine snails [23], [24]. Although the three snails all belong to the same family (Hydrobiidae), the differences in shell architecture observed across species likely evolved independently [25]. The cichlid fish *Herichthys minckleyi* (hereafter the fish) exhibits two alternative pharyngeal jaw morphologies (Fig. 2, [26]–[28]). In “papilliform” fish, the pharyngeal jaws are gracile, exhibit small muscles, and have tiny pointed teeth that allow them to shred plant material, but this morphotype is not able to crush the three snails [29]. Alternatively, “molariform” fish possess enlarged flattened teeth and robust pharyngeal muscles and are extremely proficient at crushing these three snails [29]. These two alternative morphotypes interbreed [30] and are present in all bodies of water where the three snail species occur [31]. It still unclear whether the presence of molariform teeth is determined genetically or is a plastic response to environmental conditions [32]. However, enlarged teeth can develop in the absence of snails [27] and the similarity in jaw morphology among individuals raised in the laboratory is explained by genetic similarity rather than diet [33], suggesting that this trophic polymorphism likely has a genetic basis. Shell crushing resistance is moderately heritable in at least one species of snails, *Mexipyrgus churinceanus* (unpublished data), suggesting that coevolution between shell strength and pharyngeal jaw morphology in the predatory fish is possible.

The fish likely exerts strong predation pressure on the snails. Molariform fish routinely exploit the three species of snails that are the most common snails in Cuatro Ciénegas [29], [34]. Previous analyses of gut contents in this fish species have shown that about 35% of the diet is composed of snails and 35% of plant material [27], although snails were basically found only in the guts of molariforms [29], [35]. In general, the relative abundance of prey items in the guts seems to reflect the high abundance of snails and aquatic macrophytes in the area [17], [18], [34] and the relatively low abundance of other food items such as aquatic arthropods [19], [32], [35], [36]. The snail species most commonly found in stomach contents was *Mexipyrgus churinceanus* (57.0% of snails crushed), followed by *Mexithauma quadripaludium* (29.7%), and *Nymphophilus minckleyi* (12.5%); a few other rare snail species accounted for the remaining percentage [35]. These three commonly consumed species are segregated by micro-habitat, permitting the fish to exploit particular species by foraging in particular micro-habitats. *Mexipyrgus churinceanus* is generally found only in soft sediment that is the most common substrate in most pools, *Mexithauma quadripaludium* is common on travertine substrate and rarely encountered on soft sediment or aquatic vegetation, while *Nymphophilus minckleyi* is most common on aquatic vegetation [34]. Although the proportion of snails in the gut seems to match the natural proportion of snails in the habitat, data on snail relative abundance suggest this is not necessarily the case. The relative abundance estimates of the three snail species collected from a single pool show that *Mexipyrgus churinceanus* is the most common species (78%), *Mexithauma quadripaludium* is intermediate in abundance (19%) while *Nymphophilus minckleyi* is the least abundant (3%) [37]. Hence, the fish seems to exploit the latter two species more often than expected given their relative abundances. The gut content data in conjunction with the micro-habitat and snail abundance data suggest that the fish predates heavily on the three species of snails included in this study, and that it preferentially exploits particular species.

Crushing resistance is likely the most functionally important component of defense against molariform fish in Cuatro Ciénegas. The three species of snails included in this study posses opercula [17] that cover and protect their digestible body from the outside environment [38], so the fish must crush the shell to kill and digest the snail [29], [39]. Both, experimental and field data suggest that only molariforms are able to effectively crush and feed upon snails in Cuatro Ciénegas [29], [35]. Crushing resistance increases with shell length in the three species of snails, and the snails become virtually invulnerable to crushing predation when they reach their upper shell length range at about 7mm [29]. Before reaching this size, *Mexipyrgus churinceanus* snails may be more susceptible to predation, because their shells are apparently easier to crush than the shells of snails of the other two species of similar size. In the only pool in which crushing resistance has been examined in all three species, both *Mexithauma quadripaludium* and *Nymphophilus minckleyi* had substantially stronger shells than *Mexipyrgus churinceanus* [29]. Additional studies on the crushing resistance of the three species across replicated communities are necessary to better understand the variation in anti-predator phenotypes that each species exhibits across the valley of Cuatro Ciénegas.

Previous studies suggest that current interactions among molariforms and snails have played a role in determining current shell strength within a community. In *Mexipyrgus churinceanus*, crushing resistance exhibited striking variation among populations and greater resistance to crushing was associated with the local frequency of molariform fish [18]. *Mexipyrgus churinceanus* has been present in the Cuatro Ciénegas valley for at least 2.5 million years and exhibits substantial sequence divergence between the drainages located on the western and eastern side of the Sierra, as well as between these two drainages and the Río Mesquites drainage [20]. However, neither genetic nor geographic distance explained spatial variation in *Mexipyrgus churinceanus* crushing resistance, supporting the conclusion that spatial variation in this trait is more likely due to the local abundance of molariform fish rather than phylogeographic structuring, (i.e. isolation by distance and geographic barriers to gene flow) [18]. The relationship between molariform frequency and crushing resistance in the other two snail species has not been evaluated. If shell strength does change as a local response to predation by molariform fish, we might expect similar correlations between molariform frequency and crushing resistance for all three species. Elucidating the patterns of genetic differentiation in these other two species of snails would help to determine whether spatial variation in shell crushing resistance can be at least partially explained by population genetic structure [40] or whether variation is due mostly to local factors as seems to be the case in *Mexipyrgus churinceanus*. To evaluate the potential contribution of genetic isolation to the spatial variation in crushing resistance it is particularly important to estimate levels of genetic differentiation at the population level for the three snails and the fish. Gene flow can increase or decrease the degree and rate of local adaptation depending upon heterogeneity in the environment. Gene flow has been shown to promote local adaptation when predator-prey interactions occur in a homogeneous environment, while it can limit local adaptation in heterogeneous environments [41]. The latter seems to be the case in Cuatro Ciénegas given the large difference in macrophyte density that exists among pools and rivers systems in this valley [18]. For this reason, we predict that substantial gene flow within any of these species would make local adaptation less likely in our study site. It is also critical to determine over what timeframe the fish has been interacting with the three snail species. If the fish has been interacting for a long period with the snails, local processes would have had more time to heavily influence snail defenses and/or the frequency of the fish morphotypes, making local adaptation more likely.

We examined replicate communities of these four species that span their entire geographic distribution and evaluated whether their phenotypic and genetic patterns are consistent with the geographic mosaic theory of coevolution. As stated above, the geographic mosaic theory predicts that populations should differ in the traits shaped by an interaction. To test this prediction, we ascertained if resistance to crushing varied significantly among populations within each snail species. Theory on coevolutionary dynamics in multispecies trophic interactions also predicts evolutionary shifts in predatory structures and prey defenses mediated by alternations in the preference of the predator for particular prey [6], [15], [42]. For instance, predatory intensity could cause the waxing and waning of prey defenses at different rates among species [15]. We predicted that if this coevolutionary alternation occurs crushing resistance rankings among snail species would be inconsistent across communities. Gene flow within geographic mosaics is also expected to drive local adaptation and trait remixing in both predator and prey species [41], and could explain phenotypic patterns observed among populations. We examined the potential influence of gene flow on local adaptation in this system in two ways. First, we estimated temporal and spatial patterns of genetic divergence among populations in each of three snail species and its fish predator. Then, we tested whether snail populations that were closer geographically and/or genetically were also more similar in crushing resistance and relative frequency of molariform individuals. Finally, to test for a general pattern of trait matching, we tested whether crushing resistance was correlated with the relative frequency of molariform fish across snail populations. If the proportion of molariform fish relative to papilliforms changed proportionally to average shell hardness across communities this would be a pattern consistent with trait matching across the mosaic. A lack of correlation would indicate trait mismatching in nearly all communities and not support the geographic mosaic theory of coevolution in this system.

## Methods

### Sampling procedures

Crushing resistance of *Mexipyrgus churinceanus*, *Mexithauma quadripaludium*, and *Nymphophilus minckleyi*, was measured in eleven localities nested within the three main natural drainages of Cuatro Ciénegas: pools and rivers that drain towards the western side of the Sierra, pools and rivers connected to the Río Mesquites drainage located on the eastern side of the tip of the Sierra, and isolated pools that drain towards the southeastern side of the Sierra (Table 1, Fig. 1, Table S1). Only adult snails in the upper shell length range of these relatively small species were collected to maximize the probability of finding differences in crushing resistance among sites based on differences in predation pressure (i.e. only snails that have survived predation to the point of reaching a large size were sampled). While this sampling is biased, it is equally biased among all of the taxa, which allows us to study the potential effect of predation across species. Measurements for *Mexithauma quadripaludium* and *Nymphophilus minckleyi* (n = 20 for each population) were collected over two weeks in August 2003. Crushing resistance in *Mexipyrgus churinceanus* was measured from snails collected in July 2001 from the same localities. Except for Los Remojos Negro (*n* = 12), Tío Cándido (*n* = 24), and Mojarral Este Baja (*n* = 30), twenty *Mexipyrgus churinceanus* were collected from each location.

**Table 1:** Average (95% Confidence Intervals) crushing resistance (in Newtons) uncorrected by shell length for populations of *Mexipyrgus churinceanus* (Mc), *Mexithauma quadripaludium* (Mq) and *Nymphophilus minckleyi* (Nm).

Location	Mc	Mq	Nm
Juan Santos (JS)	65.33 (58.47, 72.19)	90.46 (78.19, 102.74)	105.69 (94.74, 116.64)
Churince (CH)	79.45 (72.52, 86.38)	109.18 (96.92, 121.43)	109.40 (98.32, 120.48)
Mojarral Este Baja (MEB)	89.81 (84.19, 95.42)	110.46 (98.15, 122.77)	134.45 (123.049, 145.41)
Los Remojos Negro (LRN)	65.76 (56.94, 74.59)	112.24 (99.98, 124.49)	102.99 (92.06, 113.92)
Los Remojos Blanco (LRB)	70.53 (63.44, 77.62)	117.76 (105.48, 130.03)	115.85 (104.90, 126.80)
Tierra Blanca (TB)	108.38 (99.96, 116.81)	122.60 (110.35, 134.86)	125.20 (113.73, 136.67)
Mojarral Este Alta (MEA)	70.94 (62.93, 78.95)	123.97 (111.70, 136.25)	108.84 (97.79, 119.88)
Tío Cándido (TCS)	101.29 (94.96, 107.63)	126.11 (113.82, 138.39)	128.86 (117.90, 139.83)
Mojarral Oeste (MO)	47.42 (36.11, 58.73)	130.25 (117.97, 142.52)	129.96 (118.82, 141.11)
Río Mesquites (RM)	98.48 (90.80, 106.15)	136.76 (124.26, 149.26)	117.31 (106.37, 128.25)
North Tío Cándido (TCN)	108.22 (100.43, 116.01)	139.89 (127.53, 152.25)	142.36 (131.39, 153.32)

The snails were placed in water, transferred to the laboratory, and crushed within 4 hours of collection. As is common for many organismal traits [38], [43], crushing resistance of the three snails has been shown to scale strongly with their size [29]. Therefore, the length from the shell apex to the bottom of the aperture of each snail was measured to the nearest 0.1 mm with calipers with the goal of correcting crushing resistance by shell length when populations were statistically compared. Once brought back to the laboratory, the snails were crushed between two force plates of a Chatillon DFM50 force gauge with an automated Chatillon LTMCM-6 stand. The mobile force plate was set at 2.54 cm/min crushing speed. The force in Newtons needed to crush the snail at the time of shell failure was recorded for each snail.

### Spatial variation in crushing resistance

Shell lengths (mm) and crushing forces (Newtons) were log transformed for analyses to linearize the relationship between these two variables when simultaneously analyzed, as in previous studies [18], [29]. We compared absolute and size-adjusted crushing resistance values using Analysis of Variance (ANOVA) and Analysis of Covariance (ANCOVA), respectively, to determine if shell strengths were significantly different among populations of a species. Following [44], we used ANCOVA models for size correction when there is only one independent variable and one covariate. We fit a linear model that included crushing resistance as the dependent variable, population as a fixed effect, and shell length as covariate using the linear model function “lm” in R 2.12.0 [45] (all statistical analyses were performed in R unless stated otherwise). Post-hoc multiple comparisons of means were conducted using Tukey-Kramer tests in the R package multcomp [46].

### Comparisons of crushing resistance among snail species

Within each of our eleven sites, we also examined whether species were different in size-adjusted crushing resistance. We fit linear models (one for each community) that included crushing resistance as the dependent variable, shell length as a covariate (both log transformed), and species as a fixed effect. Post-hoc multiple comparisons of the means were also conducted using Tukey-Kramer tests.

### Temporal and spatial patterns of DNA divergence and gene flow

For genetic analyses of the snails, we sampled ten of the eleven sites mentioned above, that all contained the three snail species and the fish species. For the fish, we were only able to collect samples in eight of those localities (Table S1). Samples were collected in July 2001 and in August 2003. All snail specimens were frozen in liquid nitrogen and then stored at –80°C. In the snails, DNA was extracted from foot tissue using the Qiagen Dneasy Plant Mini Kit™ to minimize loss of DNA due to mucopolysaccharides. For *Mexipyrgus churinceanus*, we amplified and sequenced a 710 bp fragment of mitochondrial cytochrome *b* (see [20] for PCR and sequencing details). These primers did not work well in the other two species, so for *Mexithauma quadripaludium* and *Nymphophilus minckleyi,* we sequenced 634 bp and 629 bp respectively of mitochondrial cytochrome *c* oxidase subunit I (COI) with primers used with other hydrobiid snails [25]. Cytochrome *b* and COI have similar levels of sequence variation and are equally useful in recovering intrageneric phylogenetic relationships in invertebrates [47]. Mitochondrial COI amplifications were performed in 50 *µ*L solutions containing 10 mM Tris (pH 8.3), 50 mM KCl, 5.5 mM MgCl_2_, each dNTP at 200 mM, 30 pmol of each primer, 1–2 µl of undiluted template DNA, and 2 units of Taq Polymerase. The PCR cycling parameters were 60 seconds at 92°C, 50 seconds at 55°C and 2 minutes at 72°C, for 40 cycles followed by 72°C for five minutes. Amplification products were purified with Wizard PCR Preps™ (Promega, Madison, WI) or GeneClean™ (QBIOgene, Irvine, CA), and sequenced on either an ABI 377 or 3100 automated sequencer (Applied Biosystems, Foster City, CA). Sequences were aligned using CLUSTAL W [48]. In the fish, total genomic DNA was isolated from axial muscle using Puregene© extraction. A 1 µl aliquot of this solution was used to provide a DNA template for polymerase chain reaction (PCR). The entire cytochrome *b* gene was PCR amplified using primers and protocols outlined in [49]. Positively amplified DNA was then purified using an enzymatic combination of 1 µl of Exonuclease I and 1 µl shrimp alkaline phosphatase per 10 µl of PCR product. All sequences were deposited in GenBank (GU321684 - GU321898).

We evaluated whether population were differentiated at mitochondrial genes in the four species using Analysis of Molecular Variance (AMOVA). We specifically tested whether sequence variation among populations was greater than variation within populations for each species. Pair-wise fixation indices (*F*_ST_) were calculated to estimate the magnitude of differentiation between pairs of populations, which provided an idea of how different the populations are from each other. Statistical difference from zero was assessed for each index. All analysis were conducted in ARLEQUIN [50].

We reconstructed gene genealogies using BEAST 1.4.8 [51] to examine temporal divergences in the gene tree of each snail species and the fish predator and estimate the time to the most recent common ancestor (tMRCA) for each species. All samples were analyzed without taking into account the drainage or site where they were collected. We selected the best fitting, least-parameter rich model of sequence evolution based on hierarchical likelihood-ratio tests performed in MODELTEST [52]. For *Mexipyrgus churinceanus*, we used a GTR + G model of sequence evolution with clock rate of 0.010 based on 2% sequence divergence per million years [53]. For *Nymphophilus minckleyi* and *Mexithauma quadripaludium*, we used a clock rate of 0.009 based on other hydrobiid snails [54] under an HKY + G and HKY model of sequence evolution, respectively. For the fish, we used an HKY model of sequence evolution with a clock rate of 0.005 [55]. For each analysis, there were 10,000,000 steps in the MCMC chain using a UPGMA as the starting tree and a burn-in of 1,000,000. The effective sample size (the number of effectively independent draws from the posterior distribution to which the Markov chain is equivalent) for each parameter exceeded 200 in nearly all cases. Posterior probabilities and tMRCA were estimated for all nodes in the resulting trees using TreeAnnotator [51].

We complemented our analysis by estimating the magnitude of gene flow with the program IMa2 2.0 [56], [57]. This program implements a recently developed Bayesian method of isolation with migration for the analysis of divergence of multiple closely related populations, using a tree with population splitting events ordered in time. It was not possible to estimate levels of gene flow among populations due to the low sample size within each population. However, we were able to estimate levels of gene flow among the three drainages for each species, which may reflect potential levels of gene flow among populations in each generation. The method employs Markov chain Monte Carlo simulations of gene genealogies to estimate migration rates in units of the effective number of migrant gene copies per generation, which permits likelihood-ratio tests and estimates of confidence intervals. Following the program manual, we used a HKY model of substitution and set our initial priors as multiples of the population mutation rates (i.e. 4N_e_u). We estimated the effective population size (N_e_) from the data using ARLEQUIN. Specifically, we first derived estimates of genetic diversity, θ ( = 2N_e_u), for each species using the Watterson method [58] to provide an estimate of ancestral diversity. We then obtained estimates of ancestral N_e_ from the relationship θ  = 2N_e_μ using mutation estimates based on the molecular clocks given above. We used 20 heated chains with 1,000,000 trees sampled with a burn-in of 20,000 trees. The procedure was repeated at least three times with different random seeds on each dataset to confirm the consistency of the results. Effective sample sizes exceeded 200 in all cases. The phylogenetic trees were obtained for each species as described above. The genealogy obtained for *Nymphophilus minckleyi* differed from the genealogies obtained for *Mexipyrgus churinceanus* and *Mexithauma quadripaludium* (see below) and for this reason we ran separate analyses using each genealogy, with similar results. We also used both genealogies in the analysis of the fish.

### Effect of geographic and genetic distance

Following [18], we used Moran's I to test for autocorrelations in shell crushing resistance (both size-adjusted and unadjusted) and tested whether the correlations changed as a function of geographic and/or genetic distance. We repeated the analyses for the frequency of molariform fish. Moran's I is a measure of autocorrelation, with positive autocorrelations indicating that the similarity in a trait is related to how geographically-proximate and/or genetically-similar populations are. To obtain size-adjusted values of crushing resistance that could be analyzed using Moran's I, we assumed that the shell length measurements were relatively exact compared to the force estimates, and fit a linear least squares regression of shell length versus crushing resistance (both variables were log transformed) to generate “sized-adjusted” residuals for each species separately. We obtained the relative frequency of molariforms in nine of the eleven populations described above. Frequencies were obtained from Johnson et al. [31], and previously unpublished data from the Tierra Blanca site, all collected between 2000 and 2003. All estimates of percentage molariform were based on at least 20 individuals from a population. Molariform fish vary in the number of molariform teeth on their pharyngeal jaws, but the presence of at least one large flattened tooth on the lower pharyngeal jaw allows them to crush snails [18], [29]. We used this criterion to classify the fish as either papilliforms or molariforms. The relative frequency of molariform fish in a population was estimated as the percentage of molariform individuals relative to papilliforms in a sample from that population.

We tested for significant autocorrelation for linear and genetic distances in the program PASSaGE, applying a Bonferroni correction because of the multiple comparisons made [59]. We used a GPS hand-held unit to determine latitude and longitude of each population and then calculated the surface distance between them. We calculated the average genetic distance between all pairs of populations for each species using Kimura 2-parameter corrected sequence divergence of the COI gene. First, a matrix of pairwise distances among samples was constructed using ARLEQUIN, and then we obtained an average for each population pair.

### Relationship between crushing resistance and molariform frequency

For each snail species, we tested for correlations between sized-adjusted crushing resistance and molariform frequency in each population, using mean values for both variables. We only used the nine populations for which we had data on both molariform frequency and shell crushing resistance, as described above. We first confirmed that the relationship between the three variables was linear and used the method described by [60] to calculate the correlation between crushing resistance and molariform frequency while keeping shell length constant (i.e. partial correlation). Shell length and crushing resistance were log transformed. The method also estimates the statistical significance of the correlation coefficient. The R code for this method can be found at http://www.yilab.gatech.edu. We used partial residual plots to illustrate the partial correlations following recommendations in the literature [61]. Partial residual plots were made in R using the “crPlots” function in the “car” package [62].

## Results

### Spatial variation in crushing resistance

**Figure 3:**
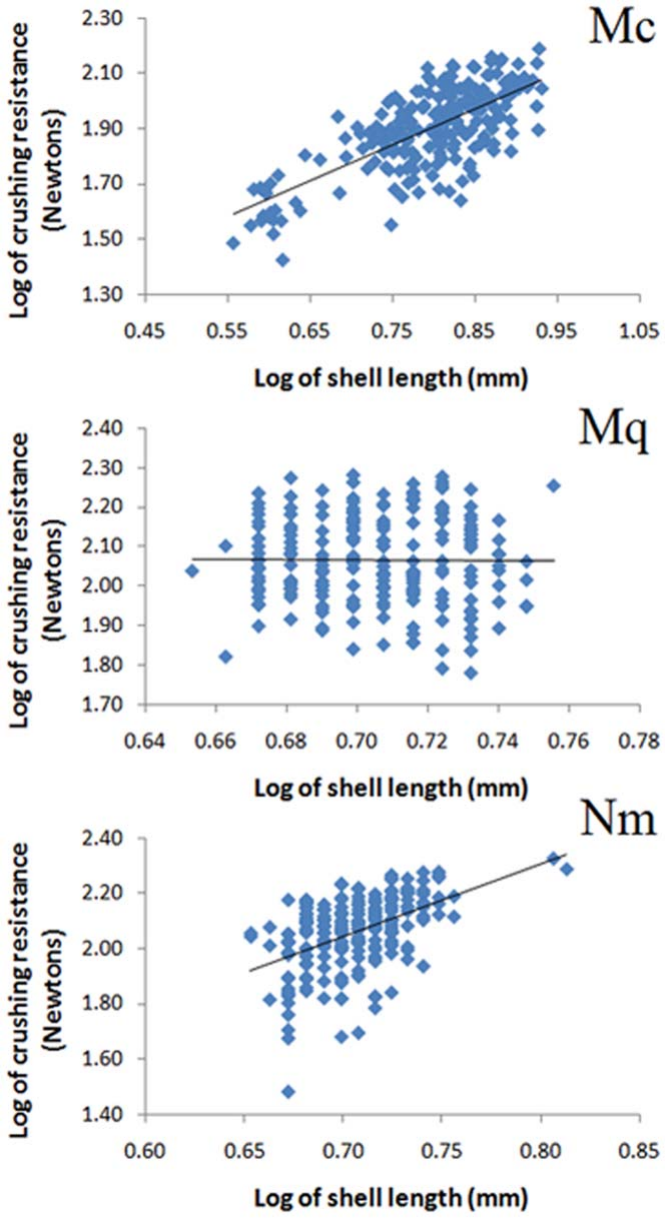
Relationship between shell length and crushing resistance (both log transformed) in *Mexipyrgus churinceanus* (Mc), *Mexithauma quadripaludium* (Mq), and *Nymphophilus minckleyi* (Nm). The best linear fit is shown for illustrative purposes (solid black line).

Average crushing resistance spanned a range of approximately 50 N among populations for very similarly sized snails (about 5 mm) of the same species (Table 1). Differences among populations were significant in each species (ANOVA: F_10, 215_ = 42.7, P<0.0001 for *Mexipyrgus churinceanus*, F_10, 209_ = 5.05, P<0.0001 for *Mexithauma quadripaludium* and F_10, 210_ = 6.55, P<0.0001 for *Nymphophilus minckleyi*). Post-hoc multiple comparison revealed that most populations differed from each other in the case of *Mexipyrgus churinceanus*, while only some populations differed from each other in the other two species (Table S2). In general, localities with low average crushing resistance, such as Juan Santos and Churince, were significantly different from places with high average crushing resistance, such as the two Tío Cándido sites and Tierra Blanca. Crushing resistance varied with shell length in *Nymphophilus minckleyi* and *Mexipyrgus churinceanus* but not in *Mexithauma quadripaludium* (Fig. 3). Crushing resistance varied among populations when adjusted by shell length in the former two species (ANCOVA: F_10,214_ = 14.8, P<0.0001 for *Mexipyrgus churinceanus*; F_10,209_ = 4.9, P<0.0001 for *Nymphophilus minckleyi*). The differences detected using multiple comparisons with size-adjusted crushing resistance values resembled those found with raw values of crushing resistance (Table S2). Interestingly, several pools separated by less than 1 km from each other and located in the same drainage, such as Mojarral Este Alta and Mojarral Este Baja had snails with different size-adjusted shell strength (especially for *Mexipyrgus churinceanus* and *Nymphophilus minckleyi*). On the other hand, snails located in different drainages and far away from each other, such as Churince and Los Remojos Blanco exhibited very similar crushing resistance in these same species (Tables 1 and 2, Fig. 1).

**Table 2:** Average (95% Confidence Intervals) crushing resistance (in Newtons) adjusted by shell length for snail populations.

Community	Mc	Mq	Nm
JS	63.1 (53.4–74.6)	89.1 (79.0–100.4)	99.4 (87.3–113.2)
CH	68.8 (60.1–78.9)	114.5 (104.1–126.0)	106.5 (96.1–118.1)
MEB	88.8 (79.2–99.6)	108.1 (96.5–121.0)	135 (120.4–151.4)
LRN	63.9 (48.2–84.8)	108.9 (94.4–125.5)	99.2 (86.0–114.4)
LRS	66.6 (58.0–76.6)	115.8 (103.4–129.6)	117.4 (106.0–130.0)
TB	77.5 (62.8–95.8)	148.4 (128.0–172.1)	159.2 (142.0–178.4)
MEA	65.0 (56.3–75.0)	120.6 (104.9–138.6)	96.4 (83.4–111.5)
TCS	92.6 (76.3–112.3)	129.5 (111.2–150.8)	135.8 (119.1–154.8)
MO	42.1 (34.1–52.0)	120.3 (103.5–139.8)	116.7 (103.0–132.2)
RM	67.0 (52.2–85.9)	166.4 (140.3–197.4)	134.1 (116.5–154.3)
TCN	96.0 (76.2–120.9)	145.0 (127.4–164.9)	146.4 (125.7–170.6)

Average crushing resistance values were calculated for the mid value of same shell length in each location using the R package “effects” [85]. Abbreviations as in Table 1.

### Comparisons of crushing resistance among snail species

*Mexipyrgus churinceanus* showed the lowest size-adjusted crushing resistance of the three species in all communities. *Mexithauma quadripaludium* and *Nymphophilus minckleyi* were similar in size-adjusted crushing resistance in most communities (Table 2, Table 3).

**Table 3:** Analysis of covariance of shell crushing resistance by snail species using shell length as covariate.

Location	F	DF	P	Mn vs Nm	Mn vs Mc	Nm vs Mc
JS	6.49	2,56	0.003	0.315	0.014	0.002
CH	13.68	2,56	<0.0001	0.433	<0.001	<0.001
LRN	4.43	2,47	0.017	0.520	0.013	0.048
LRB	17.52	2,56	<0.0001	0.975	<0.001	<0.001
MEA	19.59	2,56	<0.0001	0.079	<0.001	0.001
MEB	11.22	2,66	<0.0001	0.004	0.103	< 0.001
MO	23.05	2,55	<0.0001	0.897	<0.0001	<0.0001
RM	11.35	2,56	<0.0001	0.018	<0.001	<0.001
TB	13.39	2,57	<0.0001	0.538	<0.0001	<0.0001
TCS	3.62	2,60	0.033	0.762	0.088	0.026
TCN	3.18	2,56	0.049	0.987	0.034	0.049

P-values of multiple comparisons among snail species are shown. Species specific size-adjusted crushing resistance values are shown in Table 2. Abbreviations are shown in Table 1.

### Temporal and spatial patterns of DNA divergence and gene flow

Summary statistics for haplotype diversity, segregating sites and nucleotide diversity are presented as supplementary material (Table S3). The AMOVA showed that the haplotypes were not homogeneously distributed among populations in the case of the snails: 85–87% of sequence variation is due to differences among populations, while the remaining percentage is due to differences within populations. Pair-wise comparisons of *F*_ST_ values showed that the largest significant *F*_ST_ values correspond to comparisons between populations located on different drainages, although some populations located in the same drainage also showed significant levels of differentiation (especially all comparisons against Tierra Blanca within the Río Mesquites drainage), although these were generally lower than differentiation among the three major drainages (Tables 4–6). In general, *F*_ST_ values were higher for *Mexipyrgus churinceanus* and *Mexithauma quadripaludium* than for *Nymphophilus minckleyi*. In the case of the fish, the haplotypes were more homogenously distributed among populations. In this case, most of the variance in sequence variation was actually due to differences within populations (Table 7). Accordingly, most pair-wise *F*_ST_ comparisons were not significantly different from zero suggesting that fish populations in the pools of Cuatro Ciénegas are not as evolutionary independent as they are for the snails.

**Table 4:** Pair-wise *F*_ST_ values between pairs of populations of *Mexipyrgus churinceanus*.

	JS	CH	Esc	LR	LRS	MEA	MEB	MO	RM	TB	TCS	TCN
JS (W)	0.00											
CH (W)	0.00	0.00										
LR (RM)	**0.95**	**0.93**	0.00									
LRS (RM)	**1.00**	**1.00**	0.00	0.00								
MEA (RM)	**0.97**	**0.96**	0.16	**0.45**	0.00							
MEB (RM)	**0.96**	**0.94**	0.11	**0.37**	0.00	0.00						
MO (RM)	**0.94**	**0.93**	0.16	**0.34**	0.24	0.06	0.00					
RM (RM)	**0.98**	**0.97**	0.00	0.00	**0.35**	**0.30**	**0.32**	0.00				
TB (RM)	**0.98**	**0.98**	**0.72**	**0.90**	**0.79**	**0.75**	**0.71**	**0.84**	0.00			
TCS (SE)	**0.97**	**0.96**	**0.63**	**0.77**	**0.71**	**0.66**	**0.63**	**0.72**	**0.82**	0.00		
TCN (SE)	**0.98**	**0.98**	**0.59**	**0.83**	**0.70**	**0.62**	**0.57**	**0.75**	**0.83**	**0.47**	0.00	

AMOVA: variance among populations = 87.5%, variance within populations  = 12.5%. P<0.0001

Population abbreviations as in Table 1. Drainages: western (W), Río Mesquites (RM), and southeastern (SE). Significant *F*_ST_ (i.e. different from zero) are shown in bold.

**Table 5 pone-0022472-t005:** Pair-wise *F*_ST_ values between pairs of populations of *Mexithauma quadripaludium*.

	JS	CH	Esc	LR	LRS	MEA	MEB	MO	RM	TB	TCS	TCN
JS (W)	0											
CH (W)	**0.34**	0.00										
LR (RM)	**0.92**	**0.94**	0.00									
LRS (RM)	**0.93**	**0.96**	0.13	0.00								
MEA (RM)	**0.90**	**0.91**	0.20	0.19	0.00							
MEB (RM)	**0.92**	**0.93**	0.08	0.00	0.03	0.00						
MO (RM)	**0.94**	**0.97**	0.17	0.00	0.21	0.00	0.00					
RM (RM)	**0.92**	**0.93**	0.08	0.00	0.03	0.00	0.00	0.00				
TB (RM)	**0.92**	**0.93**	**0.70**	**0.74**	**0.63**	**0.67**	**0.78**	**0.68**	0.00			
TCS (SE)	**0.94**	**0.96**	**0.82**	**0.88**	**0.72**	**0.79**	**0.94**	**0.79**	**0.85**	0.00		
TCN (SE)	**0.92**	**0.94**	**0.82**	**0.87**	**0.74**	**0.79**	**0.91**	**0.80**	**0.83**	**0.92**	0	

AMOVA: variance among populations = 87.37%, variance within populations  = 12.63%. P<0.0001

Abbreviations as in Table 4. Significant *F*_ST_ (i.e. different from zero) are shown in bold.

**Table 6:** Pair-wise *F*_ST_ values between pairs of populations of *Nymphophilus minckleyi*.

	JS	CH	Esc	LR	LRS	MEA	MEB	MO	RM	TB	TCS	TCN
JS (W)	0.00											
CH (W)	0.37	0.00										
LR (RM)	0.30	0.41	0.00									
LRS (RM)	0.35	0.44	0.00	0.00								
MEA (RM)	0.07	**0.56**	0.35	0.38	0.00							
MEB (RM)	0.17	**1.00**	0.46	0.47	0.00	0.00						
MO (RM)	**0.69**	**1.00**	**0.61**	**0.59**	**0.56**	**1.00**	0.00					
RM (RM)	0.00	**0.33**	0.15	0.19	0.00	0.01	**0.42**	0.00				
TB (RM)	0.13	**0.83**	0.43	0.45	0.00	0.00	**0.83**	0.02	0.00			
TCS (SE)	**0.96**	**0.98**	**0.94**	**0.93**	**0.96**	**0.98**	**0.98**	**0.94**	**0.97**	0.00		
TCN (SE)	**0.94**	**1.00**	**0.76**	**0.73**	**0.91**	**1.00**	**1.00**	**0.83**	**0.98**	**0.98**	0.00	

AMOVA: variance among populations = 85.72%, variance within populations  = 14.28%. P<0.0001

Abbreviations as in Table 4. Significant *F*_ST_ (i.e. different from zero) are shown in bold.

**Table 7:** Pair-wise *F*_ST_ values between pairs of populations of the fish *Herichthys minckleyi*.

	CH	Esc	LR	MEB	MO	TB	TCS	TCN
CH (W)	0.00							
Esc (RM)	**0.76**	0.00						
LR (RM)	**0.45**	0.17	0.00					
MEB (RM)	0.03	**0.58**	0.14	0.00				
MO (RM)	0.23	**0.56**	0.02	0.00	0.00			
TB (RM)	0.16	0.71	0.00	0.00	0.00	0.00		
TCS (SE)	**0.83**	0.00	0.25	**0.60**	**0.58**	1.00	0.00	

AMOVA: variance among populations = 38.75%, variance within populations  = 61.25%. P<0.0001

Abbreviations as in Table 4. Significant *F*_ST_ (i.e. different from zero) are shown in bold.

**Figure 4:**
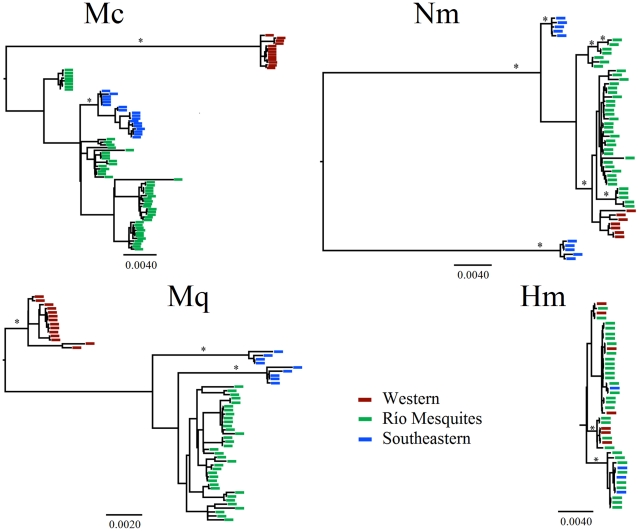
Gene genealogies for *Mexipyrgus churinceanus* (Mc) and *Herichthys minckleyi* (Hm) cytochrome *b* haplotypes, and for *Mexithauma quadripaludium* (Mq) and *Nymphophilus minckleyi* (Nm) COI haplotypes. Asterisks represent Bayesian *a posteriori* support probabilities that exceeded 0.95. Time to most recent common ancestor (myr) for each species is presented at most basal node. The scale bar at the bottom left is proportional to branch length, measured as the number of DNA substitutions per site.

Gene genealogies for all snail species were generally consistent with the AMOVA results and are presented in Figure 4. The most striking similarity between *Mexipyrgus churinceanus* and *Mexithauma quadripaludium* phylogeographic divergence was that there are two major groups corresponding to western and eastern drainages. The sequence divergence between these two drainages, as measured by the percentage of nucleotide differences per site, is 3.6% for *Mexipyrgus churinceanus* and 2.5% for *Mexithauma quadripaludium*. Within the eastern drainage, populations from upper tributaries of the Río Mesquites (Tierra Blanca) and the southeastern drainage (Tío Cándido) form well-supported groups for both *Mexipyrgus churinceanus* and *Mexithauma quadripaludium*. *Nymphophilus minckleyi* also shows considerable sequence divergence (3.2%) between the southeastern drainage (Tío Cándido) and the Río Mesquites and western drainages, although a single haplotype from Tío Cándido groups with western/Río Mesquites. In contrast with the other two species, *Nymphophilus minckleyi* shows very little sequence divergence between the Río Mesquites and western haplotypes (0.3%), with identical haplotypes found in both drainages. For the fish, there is no indication of monophyly with respect to drainages or eastern/western divisions, and several haplotypes occurs in all three drainages. The percentage of nucleotide differences per site between the western drainage and the Río Mesquites drainage is 0.19% and, similarly, there is very low divergence between the eastern and western drainages (0.2%). The mean (upper and lower 95% bounds of the highest posterior density) estimate of tMRCA was 2.46 (0.84, 4.79) myr for *Mexipyrgus churinceanus*, 2.14 (0.70, 4.12) myr for *Mexithauma quadripaludium* and 2.17 (0.72, 4.42) myr for *Nymphophilus minckleyi*. In contrast, the mean estimate of tMRCA for the fish was 0.51 (0.12, 1.05) myr. Estimates of bidirectional migration rates among drainages are lower for all three snails (a few individuals every 10 to 100 generations, not significantly different from zero) in relation to the fish predator (several individuals per generation, at least from Río Mesquites into the western drainage) (Table 8).

**Table 8:** Directional estimates of gene flow between populations (from donor to recipient) for cichlids and snails from western (W), Río Mesquites (RM), and southeastern drainages (SE) of the Cuatro Ciénegas Basin for each species estimated using Ima2 2.0.

		Donor→Recipient				
Species	W→RM	RM→W	W→SE	SE→W	RM→SE	SE→RM
Hm	3.68* (0.47, 8.63)	0.06 (0, 3.68)	0.01 (0, 5.12)	0.01 (0, 3.16)	0.01 (0, 5.59)	2.77 (0, 7.88)
Mc	0.02 (0, 3.72)	0.01 (0, 0.68)	0.02 (0, 2.36)	0.01(0, 0.66)	0.26 (0, 3.19)	0.02 (0, 3.61)
Mq	0.02 (0, 2.87)	0.02 (0, 2.95)	0.025 (0, 4.67)	0.02 (0, 2.97)	0.92 (0, 7.02)	0.25 (0, 4.2)
Nm	0.02 (0, 6.67)	0.37 (0, 5.12)	0.02 (0, 5.42)	0.02 (0, 2.82)	0.22 (0, 5.72)	0.02 (0, 3.01)

* P<0.05

The estimates and 95% profile confidence intervals (in parentheses, below) are shown for the number of immigrant females per generation. Migration rates that are significantly different from zero are indicated with asterisks. Abbreviations as in Table 1. *Herichthys minckleyi*  =  Hm.

### Effect of geographic and genetic distance

We tested for spatial and genetic autocorrelation in shell strength for each snail species (adjusted or unadjusted by size). We also tested for spatial and genetic autocorrelation in the frequency of molariform fish. The results of the analyses suggest the variation among Cuatro Ciénegas populations for these traits is not readily explained by proximity to other populations or by genetic similarity in any species (no positive autocorrelation coefficient was significantly different from zero at any distance; P>0.11 in all cases).

### Relationship between crushing resistance and molariform frequency

**Figure 5:**
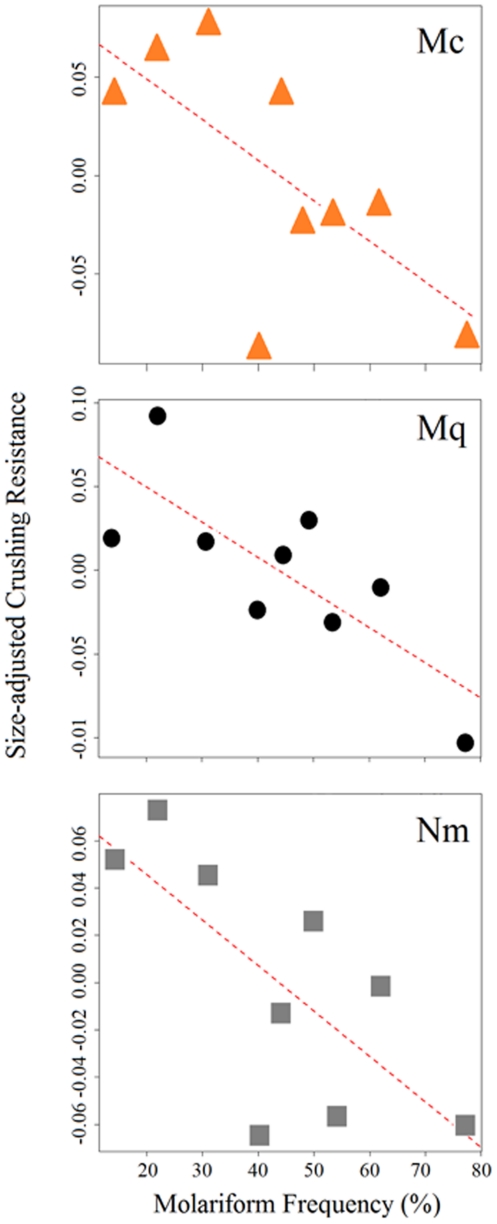
Correlation plots of population average size-adjusted crushing resistance of *Mexipyrgus churinceanus* (Mc, orange triangles), *Mexithauma quadripaludium* (Mq, black circles), *Nymphophilus minckleyi* (Nm, grey squares), and frequency of molariform cichlids. Size-adjusted crushing resistance values correspond to overall model residuals added to predicted values of crushing resistance in partial residuals plots (see text).

Relative molariform frequency ranged from 75% in Juan Santos to 14–30% in Tierra Blanca and Tío Cándido. The other habitats hovered closer to a 50% frequency of molariforms (Los Remojos Negro  = 62%, Mojarral Este  = 57%, Churince  = 48%, Mojarral Oeste  = 48%, Los Remojos Blanco  = 40%). There was a significant strong negative correlation between population molariform relative frequency and size-adjusted crushing resistance for *Nymphophilus minckleyi* (partial correlation, r =  −0.66, p = 0.03), *Mexithauma quadripaludium* (r =  −0.77, p = 0.003), and *Mexipyrgus churinceanus* (r =  −0.64, p = 0.04) (Fig. 5).

## Discussion

### Spatial variation in crushing resistance

The three freshwater snail species always required an exceptional amount of force to be crushed and their robust shells likely provide extensive defense against molariform predation. The unusual amount of force needed to crush the three snails is made clear when they are compared to other freshwater snails commonly crushed by molluscivorous fish. For example, snails consumed by predatory sunfish in temperate freshwater lakes can rarely withstand forces greater than 50 Newtons [63], [64]. The three snails from Cuatro Ciénegas easily resist two to three times this crushing force in most populations (Table 1). One possible explanation for the unusually strong shells found across populations of each snail species is that Cuatro Ciénegas is a limnologically atypical freshwater system [16] wherein shell modifications to resist crushing predation should be unusually favorable. Because of the generally high temperatures and abundant dissolved minerals, especially calcium [16], [65], calcium carbonate that snails use to construct their shells is probably not as limiting as it is in most freshwater systems [66], [67].

As predicted by the geographic mosaic of coevolution theory, crushing resistance of adult snails differed substantially among at least several populations in the three species. The large spatial variation in both unadjusted and size-adjusted crushing resistance suggests that adult snails are better defended against crushing predation in some populations than in others. An average size molariform *Herichthys minckleyi* fish can produce a compressive force of 85 Newtons, and very large molariforms can generate up to 130 Newtons [29], [35]. Hence, snails from sites where crushing resistance is higher, such as Tío Cándido or Tierra Blanca, should escape predation by most molariform fish, while snails from sites where crushing resistance is lower, such as Juan Santos and Churince should be susceptible to predation by most molariform fish (Tables 1 and 2).

### Comparisons of crushing resistance among snail species

Contrary to the predictions of the coevolutionary alternation hypothesis, crushing resistance rankings among species were consistent across communities (Tables 2 and 3). *Mexipyrgus churinceanus* was the species with the weakest shell in Cuatro Ciénegas, confirming a previous result from a single locality that found this species to be less resistant to crushing than the other two [18]. This suggests *Mexipyrgus churinceanus* should be the most susceptible species to predation by fish, as their crushing resistance rarely reaches 85 Newtons in most populations. On the other hand, *Mexithauma quadripaludium* and *Nymphophilus minckleyi* easily reach 85 Newtons in most populations (Tables 1 and 2). Previous studies show that the predatory fish crushes and ingests *Mexipyrgus churinceanus* more often than the other two snail species [35], and that it prefers to forage in soft sediment, where this species is present but the other two are rare [34], [37]. Despite the fact that the fish eats the snail *Mexipyrgus churinceanus* more frequently in absolute terms, *Mexithauma quadripaludium* and *Nymphophilus minckleyi* are consumed more often than expected given their relative abundance, while *Mexipyrgus churinceanus* is less often consumed than expected (see introduction). This could explain why *Mexipyrgus churinceanus* remains the most abundant species despite having the weakest shell and being consumed more often in absolute terms.

Previous studies have proposed that natural selection should favor predators that exploit the least defended prey more often, leading to selection for increased defenses in that prey and lower defenses in alternate prey [15]. Over time, alternation should lead to repeated cycles of switching in what prey is mostly predated and the waxing and waning of particular prey defenses [6], [15], [42]. The fact that the other two species of snails always have harder shells than *Mexipyrgus churinceanus* across sites indicates that either not enough time has passed for prey alternation to occur or that some particular physiological aspect of *Mexipyrgus churinceanus* precludes them from making their shells as hard to crush as the other two common snail species [68]. It is also possible that the other two species do not lower their defenses because it is metabolically cheap for them to produce hard shells in the unique freshwater habitats of Cuatro Ciénegas [69]. Another possibility is that the fish preferentially predates on the two harder species when they are small, and preys upon *Mexipyrgus churinceanus* the rest of the time, with the result that all species are under selection pressure to produce harder shells. Future studies can sample the distribution of sizes of the three species of snails across different relative abundances of molariform fish to test this possibility.

### Patterns of DNA divergence and gene flow and effect of genetic and geographic distances

The coalescence times for the three species of snails indicate their respective most recent common ancestors existed about 2–2.5 million years ago. Conversely, the predatory fish might have only been diversifying in the valley for about half a million years (Fig. 4). The three species of snails appear to have been in the Cuatro Ciénegas valley for approximately the same amount of time, indicating that any of the observed differences in relative shell strength are not a result of a shorter period of coevolutionary history with the fish. The analysis of population differentiation and the phylogeographic analysis reveal a long history of isolation between populations located on the eastern and western side and/or the tip of the Sierra. The same analyses indicate that populations located within the same drainage are much less differentiated, with a few exceptions (Fig. 4, Tables 4–6). Migration among populations located in the same drainage, and to a lesser extend among populations located in different drainages, probably occurs during sporadic flooding events [22]. In contrast, the fish shows little genetic differentiation among drainages and less differentiation within drainages (Fig. 4, Table 7), which is consistent with the higher levels of gene flow in comparison with the snails (Table 8). Under this scenario, shell crushing resistance should show clear geographic patterns if this trait was genetically determined and/or not strongly affected by local conditions. In the case of the fish predator, relatively high migration should dilute any local bias in the frequency of any tropic morph, so that no geographic pattern is expected to be found for molariform frequency.

### Effect of geographic and genetic distance

The geographic proximity or genetic similarity of populations did not explain inter-population variation in molariform frequency. The geographic proximity of populations did not explain inter-population variation in shell strength for any of the species. Genetic similarity also did not explain inter-population variation in crushing resistance, despite the strong historical signal of the molecular data. This result is consistent with the finding that some populations located very close to each other and within the same drainage were very different in crushing resistance, while other populations located in different drainages were not necessarily different (Table 1 and 2, Fig. 1). Clines in crushing resistance according to geographic distance and/or matching major barriers to gene flow (i.e. the Sierra, Fig. 1) can be expected if shell strength was largely affected by phylogeographic events [40]. The finding that spatial patterns in crushing resistance are not related to either geographic or genetic distance, and that therefore do not match the phylogeographic patterns recovered for the three snails species, suggests that variation in this trait might be best explained by local factors.

Producing harder shells in snails can be costly [68] (but see [69]), and it can be limited by the local availability of calcium carbonate [67], [70]. The concentration of calcium carbonate in the entire valley Cuatro Ciénegas is very high [65], [71] and is likely not a limiting factor for the production of robust shell architecture. Another potential factor that could be playing a critical role in the geographic mosaic of these species is the local abundance of aquatic macrophytes. Johnson and collaborators [18] noted that, in *Mexipyrgus churinceanus*, crushing resistance is higher in habitats dominated by aquatic macrophytes compared to habitats without aquatic macrophytes. Although most of the shell material in mollusks is composed of calcium carbonate, there is a small organic fraction, mostly proteins, that controls the formation of calcium carbonate crystals and plays key roles in determining their properties [72]–[75]. Furthermore, the deposition of calcium carbonate in the shell is affected by vitamin ingestion [76], which suggest that the amount of organic material available in the environment can determine shell strength in Cuatro Ciénegas. We hypothesize that local variation in shell strength is at least partially determined by the organic compounds that snails obtain from aquatic macrophytes in each community, a hypothesis the warrants empirical testing in the future. Interestingly, this would suggest a third trophic level could be important in setting the stage for mosaic coevolution in this system and a type of tri-trophic coevolution that has rarely been examined.

### Relationship between crushing resistance and molariform frequency

As in a previous study of the snail *Mexipyrgus churinceanus* [18], we found a low frequency of molariforms in communities with snails exhibiting high average shell strength for the three species (Fig. 5). This result suggests the possibility of coevolution between fish predatory structures and snail defenses. Snails can respond to predation pressure by developing harder shells in proportion to the magnitude of the pressure [77]–[81], i.e. local molariform frequency. However, the strong negative correlation between molariform frequency and crushing resistance appears inconsistent with this possibility.

A complementary possibility is that increases in shell strength that originally responded to the presence of molariform fish are now driving the frequency of molariforms at a local scale. The strong negative correlations (Fig. 5) in conjunction with previous results lead us to hypothesize that it may be unfavorable to be a molariform in some communities. The force necessary to crush the shell of adult snails of any species in communities such as Tío Cándido and Tierra Blanca is so high that snails at the sizes we measured are virtually invulnerable to crushing by the fish. Most adult *Mexipyrgus churinceanus* snails in those communities would require more than 100 Newtons of compressive force to be crushed at a size of about 5 mm, and the other two species exhibit even harder shells (Table 1). Snails of a larger size, which are common [17], would be impossible to crush by even the largest molariform fish [29]. Hence, it seems disadvantageous to be a molariform in communities where the shell strengths of all three species are generally high. There is a strikingly low abundance of aquatic arthropods in Cuatro Ciénegas [32], [36] leaving the fish to feed almost entirely on snails and plants in this area. Because papilliforms are able to shred aquatic macrophytes while molariforms are virtually unable to shred plant material [29], we hypothesize that papilliform fish may have an advantage over the alternative morphotype in communities dominated by aquatic macrophytes and highly durable snails. Because papilliforms are virtually incapable of crushing snails [29], molariforms may have an advantage in communities with few aquatic macrophytes and less durable snails.

### Conclusions

There are numerous examples that divergent evolution across a spatial landscape promotes diversification in offensive and defensive traits in interacting species [6], [9], [11], [82]–[84]. However, there are a limited number of studies examining potential phylogeographic effects for multiple prey species interacting with a specialized predator in multiple naturally replicated aquatic communities. We estimated that all three endemic snails have been interacting with its fish predator for a long time. There is strong spatial genetic differentiation at the regional level but not the population level for all of the snails but not for the fish. The lack of spatial autocorrelation and phylogenetic signal in shell crushing resistance and molariform relative frequency, and the reduced frequency of molariform fish in populations in which shells are extremely hard to crush suggest local biotic conditions may play an important role in shaping this putative predator-prey arms race within their small geographic range.

The patterns detected in this study are consistent with the geographic mosaic of coevolution theory. It should be noted that these patterns can be also generated by alternative process, so it is necessary to test the processes behind the patterns to provide a strong test for the theory. However, documenting the patterns in new study systems is the first step in identifying traits of importance that may warrant further study [11]. We found support for the prediction of inter-community variation not only in crushing resistance but also in molariform frequency. We also found support for the prediction of trait matching in the form of strong negative correlations between molariform frequency and crushing resistance. This suggests that the proportion of molariform fish responds proportionally to the level of defenses exhibited by the snails in each population. Deviations from a perfect correlation could be interpreted as relatively minor mismatches. Future studies could also assess whether crushing performance in the fish (i.e. pharyngeal muscle development and number of molariform teeth [29]) also matches snail crushing resistance across replicated communities. Given that gene flow has been shown to limit local adaptation in heterogeneous environments [41] such as Cuatro Ciénegas, we suggest that the limited gene flow detected among snail populations is making local adaptation of snails to biotic conditions more likely. Conversely, the substantial levels of gene flow that occur among fish populations is preventing the local fixation of any trophic morph in the fish, keeping the evolution of predatory and defensive traits highly dynamic within Cuatro Ciénegas. Given the small geographical range of the four species included in this study, the different levels of gene flow found in the fish predator and its prey, and the strong correlation found between molariform frequency and snail crushing resistance, we expect that the aquatic predator-prey network found in Cuatro Ciénegas will emerge as a model system to study networks of predator prey-interaction in geographic mosaics.

## Supporting Information

Table S1 Collection sites for DNA sequencing, number of individuals sequenced divided by drainages, and GPS coordinates for *Mexipyrgus churinceanus* (Mc), *Mexithauma quadripaludium* (Mq), *Nymphophilus minckleyi* (Nm), and *Herichthys minckleyi* (Hm).
(DOC)

Table S2 Pairwise multiple comparisons (Tukey test, P-values) of shell crushing resistance by locality, either unadjusted or adjusted for shell length for *Mexipyrgus churinceanus* (Mc), *Mexithauma quadripaludium* (Mq), and *Nymphophilus minckleyi* (Nm). Localities: Juan Santos (JS), Churince (CH), Los Remojos Negro (LRN), Los Remojos Blanco (LRB), Mojarral Este Alta (MEA), Mojarral Este Baja (MEB), Mojarral Oeste (MO), Río Mesquites (RM), Tierra Blanca (TB), Tío Cándido (TCS), North Tío Cándido (TCN). Significant values shown in bold.
(DOC)

Table S3 Number of haplotypes (n  =  number of sequenced individuals), number of segregating sites (Seg. Site) and nucleotide diversity (θ) for three geographic drainages for *Mexipyrgus churinceanus* (Mc), *Mexithauma quadripaludium* (Mq), *Nymphophilus minckleyi* (Nm), and *Herichthys minckleyi* (Hm).
(DOC)
