# Supplementary material for: Spatial Geographic Mosaic in an Aquatic Predator-Prey Network
Source: PLoS One. 2011 Jul 20;6(7):e22472. doi: 10.1371/journal.pone.0022472 (PMC3140530; doi:10.1371/journal.pone.0022472)
Supplement: Table S1 — Collection sites for DNA sequencing, number of individuals sequenced divided by drainages, and GPS coordinates for Mexipyrgus churinceanus (Mc), Mexithauma quadripaludium (Mq), Nymphophilus minckleyi (Nm), and Herichthys minckleyi (Hm). (DOC) [file pone.0022472.s001.doc]

| **Drainage/Population** | **Mc** | **Mq** | **Nm** | **Hm** | **GPS coordinates** |
| --- | --- | --- | --- | --- | --- |
| Western |  |  |  |  |  |
| Laguna Churince | 5 | 5 | 5 | 8 | 26°50.53N, 102°08.20W |
| Juan Santos | 8 | 9 | 5 | 0 | 26°53.97N, 102°08.96W |
| Río Mesquites |  |  |  |  |  |
| Tierra Blanca | 8 | 5 | 5 | 1 | 26°55.65N, 102°08.31W |
| Mojarral Oeste | 10 | 5 | 5 | 11 | 26°55.47N, 102°07.50W |
| Mojarral Este | 20 | 10 | 10 | 12 | 26°55.48N, 102°07.28W |
| Río Mesquites | 4 | 5 | 6 | 0 | 26°55.47N, 102°06.67W |
| Los Remojos | 8 | 10 | 10 | 2 | 26°55.01N, 102°06.67W |
| Laguna Escobedo | 0 | 0 | 0 | 4 | 26°53.59N, 102°05.34W |
| Southeastern |  |  |  |  |  |
| Tío Cándido N | 10 | 5 | 5 | 1 | 26°52.33N, 102°04.85W |
| Tío Cándido S | 8 | 4 | 5 | 2 | 26°52.73N, 102°05.09W |
| Total Sample Size | 81 | 58 | 56 | 41 |  |
