# Supplementary material for: Spatial Geographic Mosaic in an Aquatic Predator-Prey Network
Source: PLoS One. 2011 Jul 20;6(7):e22472. doi: 10.1371/journal.pone.0022472 (PMC3140530; doi:10.1371/journal.pone.0022472)
Supplement: Table S2 — Pairwise multiple comparisons (Tukey test, P-values) of shell crushing resistance by locality, either unadjusted or adjusted for shell length for Mexipyrgus churinceanus (Mc), Mexithauma quadripaludium (Mq), and Nymphophilus minckleyi (Nm). Localities: Juan Santos (JS), Churince (CH), Los Remojos Negro (LRN), Los Remojos Blanco (LRB), Mojarral Este Alta (MEA), Mojarral Este Baja (MEB), Mojarral Oeste (MO), Río Mesquites (RM), Tierra Blanca (TB), Tío Cándido (TCS), North Tío Cándido (TCN). Significant values shown in bold. (DOC) [file pone.0022472.s002.doc]

**Unadjusted crushing resistance**

| **Mc** | JS | CH | LRN | LRB | MEA | MEB | MO | RM | TB | TCS | TCN |
| --- | --- | --- | --- | --- | --- | --- | --- | --- | --- | --- | --- |
| JS |  |  |  |  |  |  |  |  |  |  |  |
| CH | **0.070** |  |  |  |  |  |  |  |  |  |  |
| LRN | 1.000 | 0.287 |  |  |  |  |  |  |  |  |  |
| LRB | 0.999 | 0.461 | 1.000 |  |  |  |  |  |  |  |  |
| MEA | 1.000 | 0.296 | 1.000 | 1.000 |  |  |  |  |  |  |  |
| MEB | **<0.01** | 0.492 | **<0.01** | **<0.01** | **<0.01** |  |  |  |  |  |  |
| MO | **<0.01** | **<0.01** | **<0.01** | **<0.01** | **<0.01** | **<0.01** |  |  |  |  |  |
| RM | **<0.01** | **<0.01** | **<0.01** | **<0.01** | **<0.01** | 0.369 | **<0.01** |  |  |  |  |
| TB | **<0.01** | **<0.01** | **<0.01** | **<0.01** | **<0.01** | **<0.01** | **<0.01** | 0.484 |  |  |  |
| TCS | **<0.01** | **<0.01** | **<0.01** | **<0.01** | **<0.01** | 0.171 | **<0.01** | 1.000 | 0.577 |  |  |
| TCN | **<0.01** | **<0.01** | **<0.01** | **<0.01** | **<0.01** | **<0.01** | **<0.01** | 0.632 | 1.000 | 0.728 |  |

| **Mq** | JS | CH | LRN | LRB | MEA | MEB | MO | RM | TB | TCS | TCN |
| --- | --- | --- | --- | --- | --- | --- | --- | --- | --- | --- | --- |
| JS |  |  |  |  |  |  |  |  |  |  |  |
| CH | 0.586 |  |  |  |  |  |  |  |  |  |  |
| LRN | 0.337 | 1.000 |  |  |  |  |  |  |  |  |  |
| LRB | 0.091 | 0.997 | 1.000 |  |  |  |  |  |  |  |  |
| MEA | **<0.01** | 0.820 | 0.956 | 1.000 |  |  |  |  |  |  |  |
| MEB | 0.442 | 1.000 | 1.000 | 1.000 | 0.911 |  |  |  |  |  |  |
| MO | **<0.01** | 0.390 | 0.646 | 0.942 | 1.000 | 0.531 |  |  |  |  |  |
| RM | **<0.01** | 0.092 | 0.222 | 0.589 | 0.967 | 0.155 | 1.000 |  |  |  |  |
| TB | **0.015** | 0.901 | 0.984 | 1.000 | 1.000 | 0.960 | 0.999 | 0.924 |  |  |  |
| TCS | **<0.01** | 0.721 | 0.909 | 0.997 | 1.000 | 0.840 | 1.000 | 0.988 | 1.000 |  |  |
| TCN | **<0.01** | **0.019** | 0.057 | 0.241 | 0.748 | **0.035** | 0.981 | 1.000 | 0.629 | 0.842 |  |

| **Nm** | JS | CH | LRN | LRB | MEA | MEB | MO | RM | TB | TCS | TCN |
| --- | --- | --- | --- | --- | --- | --- | --- | --- | --- | --- | --- |
| JS |  |  |  |  |  |  |  |  |  |  |  |
| CH | 1.000 |  |  |  |  |  |  |  |  |  |  |
| LRN | 1.000 | 1.000 |  |  |  |  |  |  |  |  |  |
| LRB | 0.784 | 0.694 | 0.753 |  |  |  |  |  |  |  |  |
| MEA | 1.000 | 1.000 | 1.000 | 0.733 |  |  |  |  |  |  |  |
| MEB | **0.006** | **0.003** | **0.005** | 0.574 | **0.004** |  |  |  |  |  |  |
| MO | 0.675 | 0.573 | 0.639 | 1.000 | 0.616 | 0.694 |  |  |  |  |  |
| RM | 0.743 | 0.647 | 0.710 | 1.000 | 0.688 | 0.622 | 1.000 |  |  |  |  |
| TB | **<0.001** | **<0.001** | **<0.001** | 0.190 | **<0.001** | 1.000 | 0.275 | 0.221 |  |  |  |
| TCS | **0.041** | **0.027** | **0.036** | 0.911 | **0.032** | 1.000 | 0.959 | 0.933 | 0.982 |  |  |
| TCN | **0.005** | **0.003** | **0.004** | 0.544 | **0.004** | 1.000 | 0.666 | 0.593 | 1.000 | 1.000 |  |

**Size-adjusted crushing resistance**

| **Mc** | JS | CH | LRN | LRB | MEA | MEB | MO | RM | TB | TCS | TCN |
| --- | --- | --- | --- | --- | --- | --- | --- | --- | --- | --- | --- |
| JS |  |  |  |  |  |  |  |  |  |  |  |
| CH | **0.018** |  |  |  |  |  |  |  |  |  |  |
| LRN | 1.000 | 0.096 |  |  |  |  |  |  |  |  |  |
| LRB | 0.965 | 0.475 | 0.996 |  |  |  |  |  |  |  |  |
| MEA | 0.979 | 0.631 | 0.998 | 1.000 |  |  |  |  |  |  |  |
| MEB | **<0.01** | 0.510 | **<0.01** | **<0.01** | **<0.01** |  |  |  |  |  |  |
| MO | **<0.01** | **<0.01** | **<0.01** | **<0.01** | **<0.01** | **<0.01** |  |  |  |  |  |
| RM | **<0.01** | **0.028** | **<0.01** | **<0.01** | **<0.01** | 0.866 | **<0.01** |  |  |  |  |
| TB | **<0.01** | **<0.01** | **<0.01** | **<0.01** | **<0.01** | 0.051 | **<0.01** | 0.826 |  |  |  |
| TCS | **<0.01** | **<0.01** | **<0.01** | **<0.01** | **<0.01** | 0.457 | **<0.01** | 1.000 | 0.971 |  |  |
| TCN | **<0.01** | **<0.01** | **<0.01** | **<0.01** | **<0.01** | **0.031** | **<0.01** | 0.795 | 1.000 | 0.954 |  |

| **Nm** | JS | CH | LRN | LRB | MEA | MEB | MO | RM | TB | TCS | TCN |
| --- | --- | --- | --- | --- | --- | --- | --- | --- | --- | --- | --- |
| JS |  |  |  |  |  |  |  |  |  |  |  |
| CH | 1 |  |  |  |  |  |  |  |  |  |  |
| LRN | 1.000 | 1.000 |  |  |  |  |  |  |  |  |  |
| LRB | 0.925 | 0.996 | 0.810 |  |  |  |  |  |  |  |  |
| MEA | 1.000 | 1.000 | 1.000 | 0.824 |  |  |  |  |  |  |  |
| MEB | **0.045** | 0.177 | **0.019** | 0.766 | **0.023** |  |  |  |  |  |  |
| MO | 0.082 | 0.255 | **0.041** | 0.887 | **0.037** | 1.000 |  |  |  |  |  |
| RM | 0.971 | 1.000 | 0.903 | 1.000 | 0.912 | 0.632 | 0.788 |  |  |  |  |
| TB | 0.493 | 0.836 | 0.313 | 0.999 | 0.366 | 0.995 | 1.000 | 0.996 |  |  |  |
| TCS | 0.169 | 0.458 | 0.086 | 0.965 | 0.098 | 1.000 | 1.000 | 0.911 | 1.000 |  |  |
| TCN | **<0.01** | **0.012** | **<0.01** | 0.201 | **<0.01** | 0.998 | 0.990 | 0.125 | 0.745 | 0.947 |  |
