# Supplementary material for: Spatial Geographic Mosaic in an Aquatic Predator-Prey Network
Source: PLoS One. 2011 Jul 20;6(7):e22472. doi: 10.1371/journal.pone.0022472 (PMC3140530; doi:10.1371/journal.pone.0022472)
Supplement: Table S3 — Number of haplotypes (n = number of sequenced individuals), number of segregating sites (Seg. Site) and nucleotide diversity (θ) for three geographic drainages for Mexipyrgus churinceanus (Mc), Mexithauma quadripaludium (Mq), Nymphophilus minckleyi (Nm), and Herichthys minckleyi (Hm). (DOC) [file pone.0022472.s003.doc]

|  | **Haplotypes (n)** | | | |  | **Seg. Site** | | | |  | **θ** | | | |
| --- | --- | --- | --- | --- | --- | --- | --- | --- | --- | --- | --- | --- | --- | --- |
| **Location** | **Mc** | **Mq** | **Nm** | **Hm** |  | **Mc** | **Mq** | **Nm** | **Hm** |  | **Mc** | **Mq** | **Nm** | **Hm** |
| Western | 1(13) | 6(14) | 3(10) | 2(8) |  | 0 | 6 | 2 | 1 |  | 0 | 0.0024 | 0.0011 | 0.0009 |
| Río Mesquites | 23(50) | 16(35) | 12(36) | 6(31) |  | 26 | 18 | 14 | 7 |  | 0.005 | 0.0031 | 0.0038 | 0.0021 |
| Southeastern | 7(18) | 5(9) | 5(10) | 1(3) |  | 7 | 9 | 32 | 0 |  | 0.0026 | 0.0073 | 0.0277 | 0 |
| All Drainages | 31(81) | 27(58) | 19(56) | 6(42) |  | 47 | 40 | 47 | 7 |  | 0.014 | 0.0122 | 0.0127 | 0.0021 |
